# Genome sequence of the organohalide-respiring *Dehalogenimonas alkenigignens* type strain (IP3-3^T^)

**DOI:** 10.1186/s40793-016-0165-7

**Published:** 2016-06-23

**Authors:** Trent A. Key, Dray P. Richmond, Kimberly S. Bowman, Yong-Joon Cho, Jongsik Chun, Milton S. da Costa, Fred A. Rainey, William M. Moe

**Affiliations:** Louisiana State University, Baton Rouge, LA USA; ChunLab, Inc., Seoul National University, Seoul, Republic of Korea; Department of Life Sciences, University of Coimbra, Coimbra, Portugal; University of Alaska Anchorage, Anchorage, AK USA

**Keywords:** *Chloroflexi*, *Dehalococcoidia*, Reductive dechlorination, 1,2-dichloroethane, 1,2-dichloropropane, 1,2,3-trichloropropane

## Abstract

*Dehalogenimonas alkenigignens* IP3-3^T^ is a strictly anaerobic, mesophilic, Gram negative staining bacterium that grows by organohalide respiration, coupling the oxidation of H_2_ to the reductive dehalogenation of polychlorinated alkanes. Growth has not been observed with any non-polyhalogenated alkane electron acceptors. Here we describe the features of strain IP3-3^T^ together with genome sequence information and its annotation. The 1,849,792 bp high-quality-draft genome contains 1936 predicted protein coding genes, 47 tRNA genes, a single large subunit rRNA (23S-5S) locus, and a single, orphan, small unit rRNA (16S) locus. The genome contains 29 predicted reductive dehalogenase genes, a large majority of which lack cognate genes encoding membrane anchoring proteins.

## Introduction

Strain IP3-3^T^ (=JCM 17062, =NRRL B-59545) is the type strain of the species *Dehalogenimonas alkenigignens* [1]. Currently, two pure cultures of *D. alkenigignens* have been described, namely, *D. alkenigignens* strains IP3-3^T^ and SBP-1 [1]. Both strains were isolated from chlorinated alkane- and alkene-contaminated groundwater collected at a Superfund Site near Baton Rouge, Louisiana (USA) [1]. Construction of 16S rRNA gene libraries indicated that bacteria closely related or identical to *D. alkenigignens* were present at high relative abundance in the groundwater where strains IP3-3^T^ and SBP-1 were first isolated [1].

Strains of *D. alkenigignens* possess the unique trait of growing via organohalide respiration, a process in which halogenated organic compounds are utilized as terminal electron acceptors. In particular, they are able to reductively dehalogenate a variety of polychlorinated alkanes that are of environmental concern on account of their potential to cause adverse health effects and their widespread occurrence as soil and groundwater pollutants [1–4]. In this report, we present a summary classification and a set of features for *D. alkenigignens* IP3-3^T^ together with the description of the draft genomic sequence and annotation.

## Organism information

### Classification and features

*Dehalogenimonas alkenigignens* is a member of the order *Dehalococcoidales*, class *Dehalococcoidia**,* of the phylum *Chloroflexi* (Table 1). Based on 16S rRNA gene sequences, the closest related type strains are *Dehalogenimonas lykanthroporepellens* BL-DC-9^T^ [1, 5] and *Dehalococcoides mccartyi* 195^T^ [6], with sequence identities of 96.2 and 90.6 %, respectively [1].

**Table 1:** Classification and general features of *Dehalogenimonas alkenigignens* strain IP3-3^T^ according to the MIGS recommendations [55]

MIGS ID	Property	Term	Evidence code^a^
	Classification	Domain *Bacteria*	TAS [56]
		Phylum *Chloroflexi*	TAS [57, 58]
		Class *Dehalococcoidia*	TAS [6]
		Order *Dehalococcoidales*	TAS [6]
		Family Not reported	
		Genus *Dehalogenimonas*	TAS [5]
		Species *Dehalogenimonas alkenigignens*	TAS [1]
		Type strain IP3-3^T^	TAS [1]
	Gram stain	Negative	TAS [1]
	Cell shape	Coccoid, irregular	TAS [1]
	Motility	Non-motile	TAS [1]
	Sporulation	Nonsporulating	TAS [1]
	Temperature range	18–42 °C	TAS [1]
	Optimum temperature	32–34 °C	TAS [1]
	pH range; Optimum	6.0–8.0; 6.5–7.5	TAS [1]
	Carbon source	Not reported	
MIGS-6	Habitat	Groundwater	TAS [1, 2]
MIGS-6.3	Salinity	<2 % NaCl (w/v)	TAS [1]
MIGS-22	Oxygen requirement	Obligate anaerobic	TAS [1]
MIGS-15	Biotic relationship	Free-living	NAS
MIGS-14	Pathogenicity	Non-pathogen	NAS
MIGS-4	Geographic location	Louisiana, USA	TAS [1]
MIGS-5	Sample collection	2009	IDA
MIGS-4.1	Latitude	30.590270	TAS [1]
MIGS-4.2	Longitude	−91.221288	TAS [1]
MIGS-4.4	Altitude	22 m	IDA

^a^ Evidence codes - IDA: Inferred from Direct Assay; TAS: Traceable Author Statement (i.e., a direct report exists in the literature); NAS: Non-traceable Author Statement (i.e., not directly observed for the living, isolated sample, but based on a generally accepted property for the species, or anecdotal evidence). These evidence codes are from the Gene Ontology project [59]

**Fig. 1:**
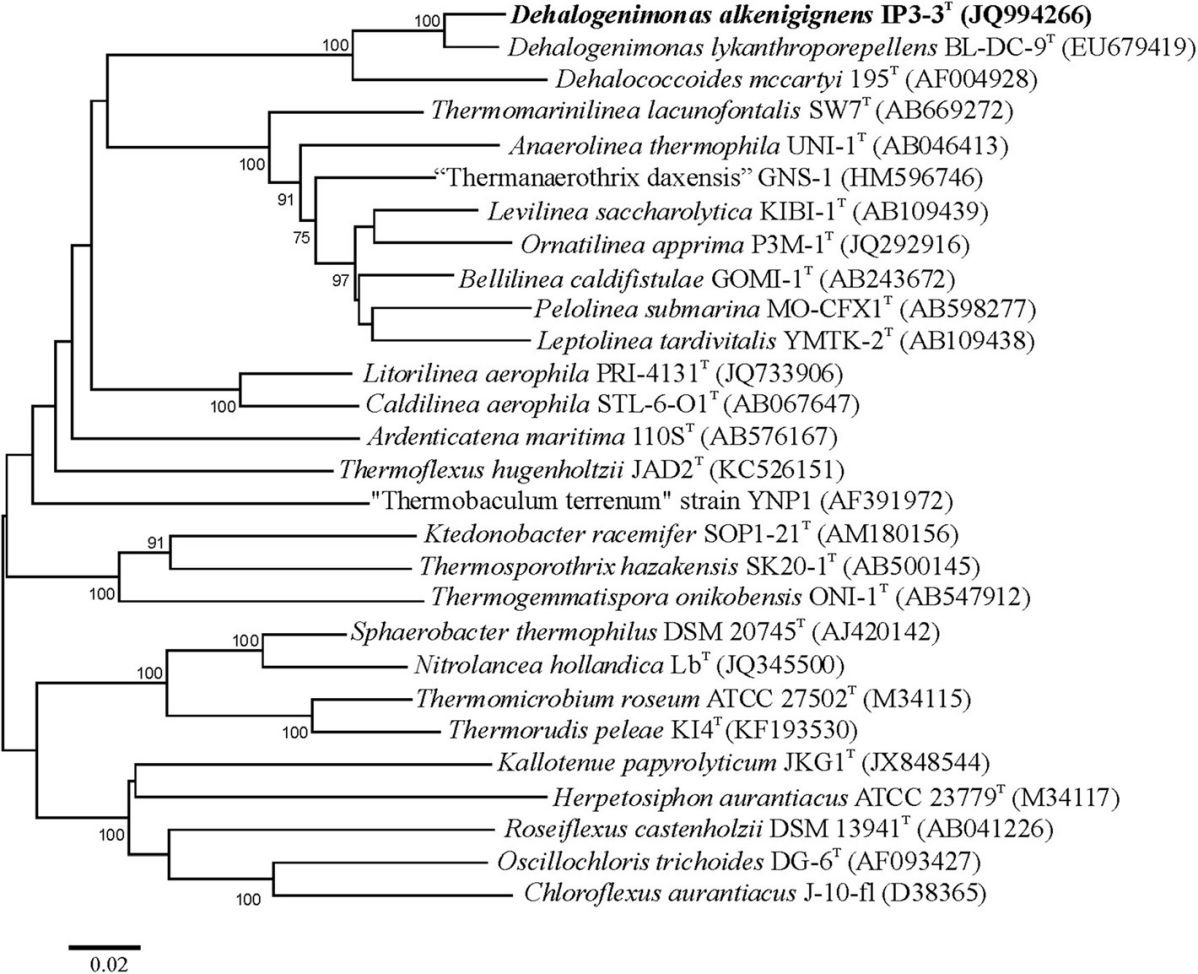
Phylogenetic tree showing the position of *D. alkenigignens* IP3-3^T^ (shown in bold) relative to the other species of the genus *Dehalogenimonas* and type species of other genera within the phylum *Chloroflexi*. The tree was inferred from 1392 aligned nucleotide positions of the 16S rRNA gene sequence using the Neighbor-Joining method within the MEGA v4.0.2 package [60]. Scale bar represents 2 substitutions per 100 nucleotide positions. Numbers at branching points denote support values from 1000 bootstrap replicates if larger than 70 %. Lineages with published genomes are: *Anaerolinea thermophila* UNI-1^T^ (AP012029), *Ardenticatena maritima* 110S^T^ (LGKN00000000), *Bellilinea caldifistulae* GOMI-1^T^ (BBXX00000000), *Caldilinea aerophila* STL-6-O1^T^ (AP012337), *Chloroflexus aurantiacus* J-10-fl^T^ (CP000909), *Dehalococcoides mccartyi* 195 ^T^ (CP000027), *Dehalogenimonas alkenigignens* IP3-3^T^ (LFDV00000000), *Dehalogenimonas lykanthroporepellens* BL-DC-9^T^ (CP002084), *Herpetosiphon aurantiacus* DSM 785^T^ (CP000875), *Kallotenue papyrolyticum* JKG1^T^ (JAGA00000000), *Ktedonobacter racemifer* SOSP1-21^T^ (ADVG00000000), *Leptolinea tardivitalis* YMTK-2^T^ (LGCK00000000), *Levilinea saccharolytica* KIBI-1 T (BBXZ00000000), *Longilinea arvoryzae* KOME-1^T^ (BBXY00000000), *Nitrolancea hollandica* Lb^T^ (CAGS00000000), *Ornatilinea apprima* P3M-1^T^ (LGCL00000000), *Oscillochloris trichoides* DG-6^T^ (ADVR00000000), *Roseiflexus castenholzii* DSM 13941^T^ (CP000804), *Sphaerobacter thermophiles* DSM 20745^T^ (CP001823), “*Thermanaerothrix daxensis*” GNS-1 (LGKO00000000), “*Thermobaculum terrenum*” YNP1 (CP001825), *Thermomicrobium roseum* DSM 5159^T^ (CP001275), and *Thermorudis peleae* KI4 ^T^ (JQMP00000000)

Figure 1 shows the phylogenetic neighborhood of *D. alkenigignens* strain IP3-3^T^ in a 16S rRNA gene based phylogenetic dendrogram. The sequence of the lone 16S rRNA gene copy in the draft genome is identical to the previously published 16S rRNA gene sequence (JQ994266).

**Fig. 2:**
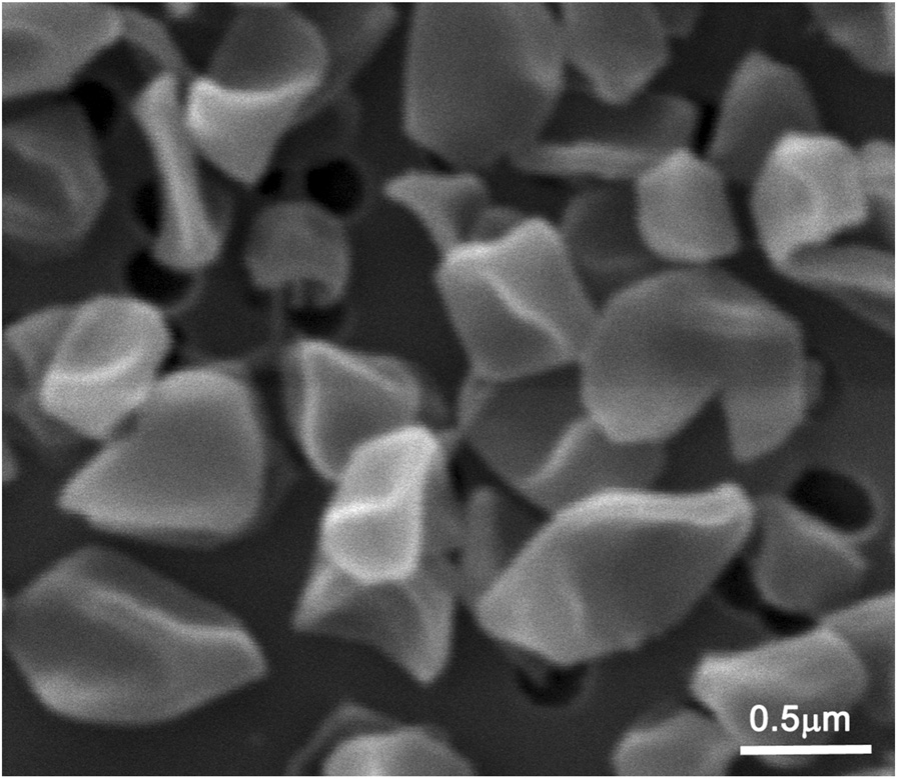
Scanning electron micrograph of cells of *D. alkenigignens* strain IP3-3^T^

The cells of *D. alkenigignens* IP3-3^T^ are Gram negative staining, non-spore forming, irregular cocci to disk-shaped with a diameter of 0.4–1.1 μm [1] (Fig. 2). The strain was isolated in liquid medium using a dilution-to-extinction approach. Growth of the strain was not observed on agar plates even after long term (2 months) incubation [1]. The temperature range for growth of strain IP3-3^T^ is between 18 °C and 42 °C with an optimum between 30 °C and 34 °C [1]. The pH range for growth is 6.0 to 8.0 with an optimum of 7.0 to 7.5 [1]. The strain grows in the presence of <2 % (w/v) NaCl and is resistant to ampicillin and vancomycin at concentrations of 1.0 and 0.1 g/l, respectively [1].

*D. alkenigignens* IP3-3^T^ is a strictly anaerobic chemotroph, coupling utilization of H_2_ as an electron donor and polychlorinated aliphatic alkanes as electron acceptors for growth. The chlorinated compounds known to be reductively dehalogenated include 1,2-dichloroethane, 1,2-dichloropropane, 1,1,2,2-tetrachloroethane, 1,1,2-trichloroethane, and 1,2,3-trichloropropane [1]. In all of the reductive dechlorination reactions characterized to date, strain IP3-3^T^ appears to exclusively utilize vicinally halogenated alkanes as electron acceptors via dihaloelimination reactions (i.e., simultaneous removal of two chlorine atoms from adjacent carbon atoms with concomitant formation of a carbon-carbon double bond) [1]. Strain IP3-3^T^ does not utilize carbon tetrachloride (tetrachloromethane), 1-chlorobenzene, chloroform, 1-chloropropane, 2-chloropropane, 1,2-dichlorobenzene, 1,1-dichloroethane, *cis*-1,2-dichloroethene, *trans*-1,2-dichloroethene, methylene chloride (dichloromethane), tetrachloroethene, 1,1,1-trichloroethane, or vinyl chloride as electron acceptors [1]. Growth is not supported by acetate, butyrate, citrate, ethanol, fructose, fumarate, glucose, lactate, lactose, methanol, methyl ethyl ketone, propionate, pyruvate, succinate, or yeast extract in the absence of H_2_ [1].

Although sufficiently high chlorinated alkane concentrations were found to become inhibitory, *D. alkenigignens* IP3-3^T^ was shown to reductively dehalogenate 1,2-dichloroethane, 1,2-dichloropropane, and 1,1,2-trichloroethane when present at initial aqueous-phase concentrations as high as 9.81 ± 0.98, 5.05 ± .29, and 3.49 ± 0.31 mM, respectively [4]. When grown in the presence of mixtures of chlorinated alkanes, preferential dechlorination of 1,1,2-trichloroethane over both 1,2-dichloroethane and 1,2-dichloropropane was observed [3]. 1,2-Dichloroethane in particular was not dechlorinated until 1,1,2-trichloroethane reached low concentrations. In contrast, *D. alkenigignens* IP3-3^T^ concurrently dechlorinated 1,2-dichloroethane and 1,2-dichloropropane over a comparably large concentration range [3].

#### Chemotaxonomic data

The major cellular fatty acids of *D. alkenigignens* IP3-3^T^ are C_18:1_*ω*9c, C_16:0_, C_14:0_, and C_16:1_*ω*9c [1]. The same fatty acids were also present in the closely related *D. alkenigignens* strain SBP-1 [1]. Cellular fatty acids present in lower proportions include C_18:0_, C_18:3_*ω*6c(6,9,12), and unidentified fatty acids with equivalent chain lengths of 11.980, 13.768, 13.937, and 15.056 [1].

## Genome sequencing information

### Genome project history

*D. alkenigignens* IP3-3^T^ was chosen for genome sequencing because it is the type strain of the species and because of the importance of organohalide respiration in the field of environmental biotechnology and bioremediation. A summary of the project information is shown in Table 2. The *D. alkenigignens* strain IP3-3^T^ genome project is deposited in the Genomes OnLine Database [7] and the genome sequence is available from GenBank.

**Table 2:** Genome sequencing project information for *Dehalogenimonas alkenigignens* IP3-3^T^

MIGS ID	Property	Term
MIGS 31	Finishing quality	Improved high-quality draft
MIGS-28	Libraries used	Three libraries: 454 Titanium standard library, 454 paired-end library (8 kb insert size), and Illumina TruSeq library
MIGS 29	Sequencing platforms	454 Titanium standard, 454 Titanium paired-end, Illumina MiSeq
MIGS 31.2	Fold coverage	42.35× (454 standard), 29.86× (454 paired-end), 583.50× (Illumina)
MIGS 30	Assemblers	Roche gsAssembler 2.6, CLCbio CLC Genomics Workbench 6.5.1
MIGS 32	Gene calling method	Prodigal
	Locus Tag	DEALK
	Genbank ID	LFDV00000000
	GenBank Date of Release	December 15, 2015
	GOLD ID	Gp0085286
	BIOPROJECT	PRJNA261058
MIGS 13	Source Material Identifier	IP3-3^T^ (=JCM 17062 = NRRL B-59545)
	Project relevance	Bioremediation, Environmental, Tree of Life

### Growth conditions and genomic DNA preparation

*D. alkenigignens* strain IP3-3^T^ (=JCM 17062, =NRRL B-59545) was cultured in liquid anaerobic basal medium [1] supplemented with 2 mM 1,2-dichloropropane. Cells were harvested from 9.9 L culture medium by centrifugation after at least 50 % of the starting 1,2-dichloropropane was dehalogenated. Total DNA was extracted using a GenElute Bacterial Genomic DNA kit (Sigma-Aldrich) following the manufacturer’s recommended protocol. Eluted DNA was concentrated using ethanol precipitation, air dried, and reconstituted in TE buffer (10 mM Tris–HCl, 0.5 mM EDTA, pH 9.0).

### Genome sequencing and assembly

The genome of *D. alkenigignens* IP3-3^T^ was sequenced using a combination of Illumina [8] and 454 technologies [9]. A total of three libraries were constructed, a 454 Titanium standard library which generated 234,711 reads (42.35-fold coverage; 78.34 Mb), a 454 Titanium paired-end libraries with insert size of 8 kb which generated 238,686 reads (29.86-fold coverage; 55.23 Mb), and an Illumina paired-end library which generated 7,147,715 reads (read length 150 bp; 583.50-fold coverage; 1079.35 Mb). Libraries were prepared using 454 standard and paired-end protocols and the Illumina TruSeq DNA sample preparation protocol, as provided by each manufacturer.

The 454 Titanium standard data and the 454 paired-end data were assembled with gsAssembler ver. 2.6 (Roche). Illumina data were assembled with CLC Genomics Workbench ver. 6.5.1 (CLCbio). Each of the resulting scaffolds and contigs were integrated using CodonCode Aligner ver. 3.7.1 (CodonCode Corporation). Also, Illumina sequencing reads were mapped to the final contigs to correct misassembles and base errors. The final assembly generated one scaffold including two contigs representing 1,849,792 bp based on 655.71× coverage of 454 and Illumina sequencing data.

### Genome annotation

Genes were identified using Prodigal [10] as part of the JGI’s microbial annotation pipeline [11] followed by a round of manual curation using the JGI GenePRIMP pipeline [12]. The predicted CDSs were translated and used to search the National Center for Biotechnology Information nonredundant database, UniProt, TIGRFam, Pfam, PRIAM, KEGG, COG, and InterPro databases. These data sources were combined to assert a product description for each predicted protein. Non-coding genes and miscellaneous features were predicted using tRNAscan-SE [13], RNAMMer [14], Rfam [15], TMHMM [16], ARAGORN [17], bSECISearch [18], and signal [19]. Additional gene prediction analysis and manual functional annotation was performed within the Integrated Microbial Genomes - Expert Review platform [20].

## Genome properties

**Fig. 3:**
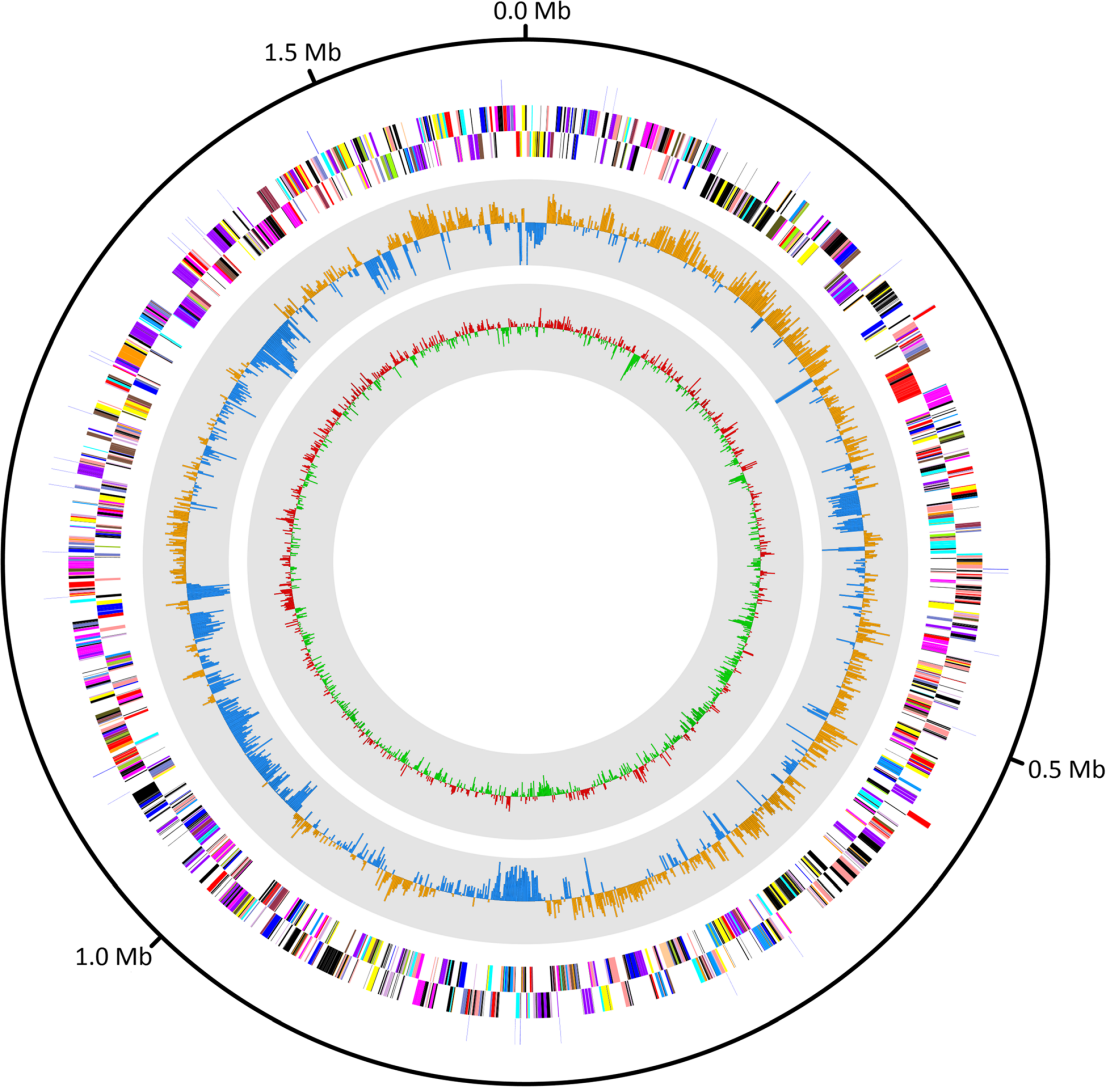
Graphical circular map of the largest contig. From the outside to the center: RNA genes (rRNAs in red and tRNAs in blue), genes on the reverse strand (colored according to the COGs categories), genes on the forward strand (colored according to the COGs categories), GC skew (where yellow indicates positive values and blue indicates negative values), GC ratio (shown in red/green, which indicates positive/negative, respectively)

The draft genome of *D. alkenigignens* strain IP3-3^T^ has a total length of 1,849,792 bp with 55.88 % G + C content (Table 3 and Fig. 3). Of the 1988 genes predicted, 1936 were protein-coding genes and 52 were RNAs. The majority of the protein-coding genes (74.9 %) were assigned a putative function, and the remaining were annotated as hypothetical proteins. The distribution of the predicted protein coding genes into COG functional categories is presented in Table 4.

**Table 3:** Genome statistics for *Dehalogenimonas alkenigignens* IP3-3^T^

Attribute	Value	% of Total
Genome size (bp)	1,849,792	100.00 %
DNA coding (bp)	1,667,990	90.17 %
DNA G + C (bp)	1,033,591	55.88 %
DNA scaffolds	1	
Total genes	1988	100.00 %
Protein coding genes	1936	97.38 %
RNA genes	52^a^	2.62 %
Pseudo genes	4	0.20 %
Genes in internal clusters	1270	63.88 %
Genes with function prediction	1489	74.90 %
Genes assigned to COGs	1164	58.55 %
Genes with Pfam domains	1505	75.70 %
Genes with signal peptides	57	2.87 %
Genes with transmembrane helices	455	22.89 %
CRISPR repeats	0	0 %

^a^ The genome contains a single large subunit rRNA (23S-5S) locus and a single, orphan, small subunit rRNA (16S) locus

**Table 4:** Number of protein coding genes of *Dehalogenimonas alkenigignens* IP3-3^T^ associated with general COG functional categories

Code	Value	%age^a^	Description
J	156	12.06	Translation, ribosomal structure and biogenesis
A	0	0.00	RNA processing and modification
K	70	5.41	Transcription
L	69	5.33	Replication, recombination and repair
B	2	0.15	Chromatin structure and dynamics
D	10	0.77	Cell cycle control, Cell division, chromosome partitioning
V	17	1.31	Defense mechanisms
T	68	5.26	Signal transduction mechanisms
M	29	2.24	Cell wall/membrane biogenesis
N	11	0.85	Cell motility
U	14	1.08	Intracellular trafficking and secretion
O	77	5.95	Posttranslational modification, protein turnover, chaperones
C	99	7.65	Energy production and conversion
G	46	3.55	Carbohydrate transport and metabolism
E	142	10.97	Amino acid transport and metabolism
F	50	3.86	Nucleotide transport and metabolism
H	91	7.03	Coenzyme transport and metabolism
I	45	3.48	Lipid transport and metabolism
P	82	6.34	Inorganic ion transport and metabolism
Q	14	1.08	Secondary metabolites biosynthesis, transport and catabolism
R	124	9.58	General function prediction only
S	66	5.10	Function unknown
-	824	41.45	Not in COGs

^a^The total is based on the total number of protein coding genes in the genome

## Insights from the genome sequence

### Comparative genomics

The draft genome of *D. alkenigignens* IP3-3^T^ is 163,282 bp larger than that of *D. lykanthroporepellens* BL-DC-9^T^ (1,686,510 bp) and 380,072 bp larger than *Dehalococcoides mccartyi* 195^T^ (1,469,720 bp). Although the genomes of *D. alkenigignens* IP3-3^T^, *D. lykanthroporepellens* BL-DC-9^T^ [21], and *Dehalococcoides mccartyi* strains [22–24] contain similar number of rRNA and tRNA encoding genes, they lack overall synteny and differ in their GC content, gene density, and percentage of sequence that encodes proteins.

BLAST comparisons of protein sets of *D. alkenigignens* IP3-3^T^ and *D. lykanthroporepellens* BL-DC-9^T^ revealed that the two strains contain 1154 protein coding genes in common (bidirectional best hits, 20-95 % identity at the predicted protein level). Bidirectional best-hit comparisons indicated that *D. alkenigignens* IP3-3^T^ contains 782 protein-coding genes with no homologs in *D. lykanthroporepellens* BL-DC-9^T^. The latter contained 566 protein-coding genes with no homologs in *D. alkenigignens* IP3-3^T^. Genome-specific genes identified in *D. alkenigignens* IP3-3^T^ and *D. lykanthroporepellens* BL-DC-9^T^ included those that encoded transposases, restriction endonucleases, acetyltransferases, permeases, reductases, hydrogenases, and dehalogenases. Some of these strain-specific genes were associated with IS elements.

Nine signature indels (insertions or deletions) specific for predicted proteins of the class *Dehalococcoidia* (which at present includes only the genera *Dehalococcoides* and *Dehalogenimonas*) were recently reported based on the results of comparative analyses of previously reported genomes [25]. Of the nine proteins in which conserved signature indels were reported as specific for the class *Dehalococcoidia* [25], all were found to be present in the predicted proteins of *D. alkenigignens* IP3-3^T^, including those for a GTP binding protein LepA (DEALK_16110), F_0_F_1_-type ATP synthase alpha subunit (DEALK_14680), imidazoleglycerol-phosphate dehydratase (DEALK_15410), glycine/serine hydroxymethyltransferase (DEALK_18820), adenylate kinase (DEALK_03090), hydrogenase formation/expression protein HypD (DEALK_04300), DNA gyrase subunit A (DEALK_05640), excinuclease ABC subunit A (DEALK_13870), and ribulose-phosphate 3-epimerase (DEALK_13610). Of the conserved signature proteins (CSPs) that were reported previously to be specific for the class *Dehalococcoidia* [25], however, several did not have homologs in *D. alkenigignens* IP3-3^T^ (DET0078, DET0236, DET0307, DET0767, DET1026, DET1283, and DET1511). Furthermore, four conserved signature proteins reported as specific for the genus *Dehalococcoides* [25] (DET0939, DET1011, DET1322, and DET1557) were found to have homologs in *Dehalogenimonas alkenigignens* IP3-3^T^ (DEALK_12980, DEALK_11520, DEALK_01350, and DEALK_19030, respectively), indicating that these proteins are not as narrowly confined to the genus *Dehalococcoides* as once thought.

The genome of *D. alkenigignens* IP3-3^T^ contains 47 tRNA genes, including those for all 20 standard amino acids as well as the less common amino acid selenocysteine. Consistent with the presence of a *selC* gene (DEALK_t00110) encoding a selenocysteine-inserting tRNA (tRNA^sec^), *D. alkenigignens* strain IP3-3^T^ also contains genes that are putatively involved in synthesis of selenocysteine (DEALK_04960-04970) and a GTP-dependent selenocysteine-specific elongation factor (DEALK_04950) that forms a quaternary complex with selenocysteine-tRNA^sec^ and the selenocysteine inserting sequence (SECIS), a hairpin loop found immediately downstream of the UGA codon in selenoprotein-encoding mRNA [26]. This complex facilitates reading through the UGA codon and incorporation of selenocysteine instead of translation termination [27]. Also consistent with the presence of the genes encoding the synthesis and incorporation of selenocysteine, *D. alkenigignens* strain IP3-3^T^ contains multiple genes encoding putative selenocysteine-containing proteins including a selenophosphate synthase (DEALK_04975) and formate dehydrogenase (DEALK_19115) that have internal in-frame UGA stop codons followed by putative SECIS elements [18].

A number of microorganisms accumulate low molecular weight organic compounds commonly referred to as “compatible solutes” that help the microorganisms survive osmotic stress but do not interfere with metabolism [28]. Ectoine is a compatible solute of many mesophilic bacteria capable of survival at high salt concentrations [28], while mannosylglycerate is a compatible solute accumulated by many thermophilic organisms [29]. Homologs of a gene encoding a bifunctional mannosylglycerate synthase (*mgsD*) are found in *Dehalococcoides mccartyi* strains (e.g., DET1363) and *D. lykanthroporepellens* BL-DC-9^T^ (Dehly_0877), an unusual occurrence for mesophilic bacteria [21, 29]. Comparative analysis revealed that *D. alkenigignens* IP3-3^T^ contains a homologous gene (DEALK_12650, 55–70 % protein identity). This expands the range of mesophilic species containing genes putatively involved in the biosynthesis of mannosylglycerate. *D. alkenigignens* IP3-3^T^, however, lacks the operon (*ectABC*) encoding putative homologs of the enzymes involved in ectoine biosynthesis and regulation that were found to be present in *D. lykanthroporepellens* BL-DC-9^T^ (Dehly_1306, Dehly_1307, Dehly_1308). The presence of these ectoine encoding genes in *D. lykanthroporepellens* BL-DC-9^T^ but not *D. alkenigignens* IP3-3^T^ may account for the ability of the former to reductively dechlorinate polychlorinated alkanes in the presence of higher NaCl concentrations than was observed for *D. alkenigignens* IP3-3^T^ [1].

### Reductive dehalogenases

Genes encoding the enzymes characterized to date that are involved in catalyzing the reductive dehalogenation of chlorinated solvents are organized in *rdhAB* operons encoding a ~500 aa protein (RdhA) that functions as a reductive dehalogenase and a ~90 aa hydrophobic protein with transmembrane helices (RdhB) that is thought to anchor the RdhA to the cytoplasmic membrane [30–41]. *D. alkenigignens* IP3-3^T^ contains several loci, accounting for 2.38 % of the genome, related to *rdhA* and/or *rdhB* genes scattered throughout the genome. The multiple *rdhA* and *rdhB* ORFs of *D. alkenigignens* IP3-3^T^ have 32–97 % and 32–43 % identities at the predicted protein level, respectively. The closest homologs for most of the *D. alkenigignens* IP3-3^T^*rdhA* ORFs (Table 5) are found among *Dehalogenimonas lykanthroporepellens* BL-DC-9^T^, *Dehalococcoides mccartyi* strains, or uncultured bacteria. A twin-arginine motif followed by a stretch of hydrophobic amino acids, was identified in the N-terminus of a large majority (27 of 29) of the predicted RdhA sequences (Table 5). Consistent with the presence of the twin-arginine sequence in the N-terminus of most of its RdhA sequences, *D. alkenigignens* IP3-3^T^ contains an operon encoding proteins that constitute a putative twin-arginine translocation (TAT) system (DEALK_04830-04860). This specialized system is involved in the secretion of folded proteins across the bacterial inner membrane into the periplasmic space [42, 43]. *Dehalogenimonas lykanthroporepellens* BL-DC-9^T^ also contains an operon encoding an analogous TAT system that is related to the TAT system of *D. alkenigignens* IP3-3^T^ (55–86 % protein identity).

**Table 5:** Characteristics of putative reductive dehalogenases (rdhA) of *Dehalogenimonas alkenigignens* IP3-3^T^

Locus tag	ORF size (bp)	mol% G + C	Protein size (aa)	TAT Signal Sequence	Fe-S binding motif #1	Fe-S binding motif #2	Cognate rdhB	Closest homolog		
								Accession/locus tag	Identity	Size (aa)
DEALK_00310	1344	44.64	447	-	CX_2_CX_2_CX_3_C	CX_10_CX_2_CX_3_C	None	DET0876	38 %	510
DEALK_00330	1584	45.58	527	+	CX_2_CX_2_CX_3_C	CX_11_CX_2_CX_3_C	None	GY50_1378	38 %	508
DEALK_01520	1566	58.88	521	+	CX_2_CX_2_CX_3_C	CX_9_CX_2_CX_3_C	None	DGWBC_1268	43 %	500
DEALK_04890	1593	50.03	530	+	CX_2_CX_2_CX_3_C	CX_8_CX_4_CX_3_C	None	DGWBC_1769	81 %	531
DEALK_05980	1515	62.44	504	+	CX_2_CX_2_CX_3_C	CX_2_CX_3_C	None	DGWBC_1268	42 %	500
DEALK_05990	1542	59.27	513	+	CX_2_CX_2_CX_3_C	CX_12_CX_2_CX_3_C	None	BAI47820.1	60 %	490
DEALK_06000	1518	58.56	505	+	CX_2_CX_2_CX_3_C	CX_12_CX_2_CX_3_C	None	BAI47820.1	59 %	490
DEALK_06060	1416	59.82	471	+	CX_2_CX_2_CX_3_C	CX_9_CX_4_CX_3_C	None	Dehly_0849	68 %	475
DEALK_06310	1422	54.57	473	+	CX_2_CX_2_CX_3_C	CX_9_CX_2_CX_3_C	None	DhcVS_1421	63 %	475
DEALK_06360	1527	58.74	508	+	CX_2_CX_2_CX_3_C	CX_12_CX_2_CX_3_C	None	DGWBC_1268	44 %	500
DEALK_07340	1398	59.73	465	+	CX_2_CX_2_CX_3_C	CX_8_CX_2_CX_3_C	None	AGY79010.1	63 %	413
DEALK_07360	1398	54.01	465	+	CX_2_CX_2_CX_3_C	CX_8_CX_2_CX_3_C	None	Dehly_1582	75 %	452
DEALK_08250	1575	52.38	524	+	CX_2_CX_2_CX_3_C	CX_10_CX_2_CX_3_C	None	X793_01190	45 %	514
DEALK_08260	1566	56.64	521	+	CX_2_CX_2_CX_3_C	CX_9_CX_2_CX_3_C	None	X793_01190	42 %	514
DEALK_08270	1518	50.00	505	+	CX_2_CX_2_CX_3_C	CX_8_CX_2_CX_3_C	None	DGWBC_1584	77 %	502
DEALK_11210	1404	56.91	467	+	CX_2_CX_2_CX_3_C	CX_8_CX_2_CX_3_C	None	Dehly_0121	69 %	469
DEALK_11290	1527	61.03	508	+	CX_2_CX_2_CX_3_C	CX_9_CX_2_CX_3_C	DEALK_11280	BAG72164.1	42 %	504
DEALK_11300	1416	56.64	471	+	CX_2_CX_2_CX_3_C	CX_2_CX_2_CX_3_C	None	AGY79025.1	75 %	367
DEALK_11330	1401	59.10	466	+	CX_2_CX_2_CX_3_C	CX_2_CX_2_CX_3_C	None	AGY79025.1	77 %	367
DEALK_11430	1575	53.14	524	+	CX_2_CX_2_CX_3_C	CX_10_CX_2_CX_3_C	None	X793_01190	44 %	514
DEALK_16100	1386	55.27	461	+	CX_2_CX_2_CX_3_C	CX_9_CX_4_CX_3_C	None	Dehly_0068	69 %	460
DEALK_16320	1401	58.82	466	+	CX_2_CX_2_CX_3_C	CX_8_CX_2_CX_3_C	None	DGWBC_0119	74 %	474
DEALK_16330	1515	61.65	504	+	CX_2_CX_2_CX_3_C	CX_9_CX_2_CX_3_C	None	DGWBC_0120	80 %	502
DEALK_17120	1449	46.79	482	+	CX_2_CX_2_CX_3_C	CX_8_CX_2_CX_3_C	None	CEP66756.1	42 %	449
DEALK_17180	849	42.84	282	-	None	None	None	Dehly_1523	92 %	340
DEALK_17200	1455	44.47	484	+	CX_2_CX_2_CX_3_C	CX_2_CX_3_C	DEALK_17210	AGS15112.1	95 %	484
DEALK_17450	1563	58.99	520	+	CX_2_CX_2_CX_3_C	CX_9_CX_2_CX_3_C	None	X793_01190	42 %	514
DEALK_17880	1641	60.69	546	+	CX_2_CX_2_CX_3_C	CX_2_CX_3_C	None	DGWBC_1268	40 %	500
DEALK_19050	1506	50.60	501	+	CX_2_CX_2_CX_3_C	CX_2_CX_2_CX_3_C	DEALK_19040	DhcVS_96	61 %	496

Two conserved motifs each containing three or four cysteine residues, a feature associated with binding iron-sulfur clusters [44], were identified near the C-terminus of 28 of the 29 predicted RdhA sequences of *D. alkenigignens* IP3-3^T^. The first of these motifs had a consistent number of cysteine residues and consistent number of amino acids between the cysteine residues (CX_2_CX_2_CX_3_C), while the second motif was variable (Table 5). If a “full-length” *rdhA* is predicted to encode a protein containing a twin-arginine sequence in the N-terminus, two iron-sulfur cluster binding motifs in the C-terminus, and an intervening sequence of ~450 aa, then *D. alkenigignens* IP3-3^T^ contains 27 such genes, a number appreciably larger than the 17 such genes found in *Dehalogenimonas lykanthroporepellens* BL-DC-9^T^ [21]. One of the non-full length *rdhA* genes (DEALK_17180) contains a predicted internal stop codon that putatively prevents complete translation of what would otherwise be a 458 aa protein containing two iron-sulfur binding clusters. *rdhA* genes with internal stop codons have been reported previously among the genomes of other organohalide respiring strains of the genera *Dehalococcoides* [24] and *Dehalobacter* [45, 46].

Within *D. alkenigignens* IP3-3^T^, only three of the *rdhA* ORFs (DEALK_11290, DEALK_17200, and DEALK_19050) have a cognate *rdhB* (Table 6). Two additional *rdhB* genes (DEALK_00250 and DEALK_05730) appear to be orphans with no cognate *rdhA* ORF. In at least one locus (DEALK_00250), it appears that transposon insertion has truncated the *rdhA* gene (annotated as pseudogene DEALK_00260). The predicted RdhB sequences of strain IP3-3^T^ each contain two or three transmembrane helices (Table 6), similar to the features of the predicted RdhB sequences of *Dehalogenimonas lykanthroporepellens* BL-DC-9^T^ and *Dehalococcoides mccartyi* strains [21, 22, 24, 47]. The predicted RdhB sequences of *D. alkenigignens* IP3-3^T^ are most closely related to the RdhB of *D. lykanthroporepellens* strain BL-DC-9^T^, *Dehalococcoides mccartyi* strain GY, and an uncultured bacterium designated as *Dehalogenimonas* sp. WBC-2 [48] (45-96 % identity at the protein level, Table 6). As was observed for *D. lykanthroporepellens* BL-DC-9^T^ [21], genes putatively involved in the regulation of *rdhAB* operons in *Dehalococcoides mccartyi* strains (e.g.*,* MarR-type or two-component transcriptional regulators [22, 24]) were not present in a majority of the *rdhA* loci of *D. alkenigignens* IP3-3^T^. Thus, it appears that regulation of *rdh* gene expression within the genus *Dehalogenimonas* may generally differ from that of the genus *Dehalococcoides*.

**Table 6:** Putative reductive dehalogenase membrane anchoring proteins (rdhB) of *Dehalogenimonas alkenigignens* IP3-3^T^

Locus tag	ORF size (bp)	mol% G + C	Protein size (aa)	TM^a^	Cognate rdhA	Closest homolog		
						Locus tag	Identity	Size (aa)
DEALK_00250	285	42.81	94	3	None ^b^	GY50_1377	45 %	91
DEALK_05730	270	58.52	89	3	None	DGWBC_0212	85 %	89
DEALK_11280	294	59.52	97	3	DEALK_11290	Dehly_1504	56 %	88
DEALK_17210	228	37.28	75	2	DEALK_17200	Dehly_1525	96 %	72
DEALK_19040	279	49.46	92	3	DEALK_19050	Dehly_0276	70 %	91

^a^ Number of transmembrane helices as predicted by TMHMM2.0 [16]

^b^ A pseudogene (DEALK_00260) upstream of the putative *rdhB* gene is predicted to encode a 33 aa fragment with high sequence identity (63 %) with the C-terminus of a putative reductive dehalogenase of *Dehalococcoides mccartyi* 195^T^ (DET0235)

The predicted RdhA protein encoded by the *rdhAB* operon comprised of DEALK_17200-17210 shares 95 % identity with the 1,2-dichloropropane reductive dehalogenases (dcpAs) recently identified in *Dehalococcoides mccartyi* strains KS and RC and 92 % identity with the related dcpA in *D. lykanthroporepellens* BL-DC-9^T^ [39]. The putative membrane anchoring protein encoded by the *rdhB* (DEALK_17210) adjacent to the *dcpA* gene is also related (92–96 % identity at the protein level) to the RdhB cognate dcpA of *D. lykanthroporepellens* BL-DC-9^T^ and *Dehalococcoides mccartyi* strains KS and RC [39]. Interestingly, the putative *dcpA* gene present in *D. alkenigignens* IP3-3^T^ had mismatches with all four primers/probes that were reported [39] for use in PCR or qPCR for detection and quantification of this gene (1 mismatch with *dcpA*-360 F, 3 mismatches with *dcp*A-1257 F, and two mismatches each with *dcpA*-1426R and *dcpA*-1449R).

The presence of insertion sequence elements adjacent to some *rdhA/rdhB* loci in *D. alkenigignens* IP3-3^T^ indicates their acquisition from an unknown host. Previous studies of *D. lykanthroporepellens* BL-DC-9^T^ and *Dehalococcoides* strains have also suggested horizontal transfer of reductive dehalogenase genes [21, 49, 50]. Additionally, the genomic region downstream of the *ssrA* gene (DEALK_tm00010) in *D. alkenigignens* IP3-3^T^ shares some synteny with the mobile genetic elements reported for vinyl chloride reductases in *Dehalococcoides* strains [49]. A 22 bp direct repeat of the 3’ end of the *ssrA* gene adjacent to one of the *rdhA* loci in *D. alkenigignens* IP3-3^T^ (DEALK_11430) suggests that integration at the *ssrA* gene may have played a role in shaping the genome of *D. alkenigignens* IP3-3^T^.

It remains to be determined if *D. alkenigignens* IP3-3^T^*rdhA* genes lacking an *rdhB* ORF downstream encode functional reductive dehalogenases and whether or how they are membrane-bound. It is possible that a non-contiguous *rdhB* (e.g., the orphan DEALK_005730) could complement one or more of the strain IP3-3^T^*rdhA* genes lacking an *rdhB* ORF downstream. Alternatively, some of these genes may encode reductive dehalogenases that are not membrane bound or that are bound by an unknown mechanism. The finding that many of the *D. lykanthroporepellens* BL-DC-9^T^*rdhA* genes lacking cognate *rdhB* genes are simultaneously transcribed during the reductive dechlorination of 1,2-dichloroethane, 1,2-dichloropropane, and 1,2,3-trichloropropane [51] suggests that *rdhA* genes lacking a cognate *rdhB* may serve a purpose. An enzyme involved in the reductive dehalogenation of tetrachloroethene by *Sulfurospirillum multivorans* (basonym *Dehalospirillum multivorans* [52, 53]) was found in the cytoplasmic fraction [54], suggesting that some reductive dehalogenases are either loosely membrane-bound or soluble entities. The same may be the case for the majority of reductive dehalogenases of *D. alkenigignens* IP3-3^T^.

## Conclusions

Genomic analysis of *D. alkenigignens* IP3-3^T^ revealed the presence of components associated with synthesis of selenocysteine-containing proteins as well as numerous reductive dehalogenase homologous genes not previously studied. As with the related species *D. lykanthroporepellens* but in contrast to other dechlorinating genera, a large majority of the reductive dehalogenase homologous genes in *D. alkenigignens* IP3-3^T^ lack apparent cognate genes encoding membrane anchoring components. The sequences of these diverse genes may aid future studies aimed at elucidating the strain’s mechanisms for transforming polychlorinated alkanes. The absence of genes encoding enzymes involved in ectoine biosynthesis in the genome of *D. alkenigignens* IP3-3^T^ may account for the strain’s inability to dehalogenate chlorinated alkanes at higher NaCl concentrations that were observed for strains of the related species *D. lykanthroporepellens*.
